# Establishment of *Biomphalaria tenagophila* Snails in Europe

**DOI:** 10.3201/eid1411.080479

**Published:** 2008-11

**Authors:** Gábor Majoros, Zoltán Fehér, Tamás Deli, Gábor Földvári

**Affiliations:** Szent István University Faculty of Veterinary Science, Budapest, Hungary (G. Majoros, G. Földvári); Hungarian Natural History Museum, Budapest (Z. Fehér); Munkácsy Mihály Museum, Békéscsaba, Hungary (T. Deli); 1 These authors contributed equally to this article.

**Keywords:** Biomphalaria tenagophila, Europe, schistosomiasis, mollusks, Planorbidae, emergence, spread, snail, intermediate host, Romania, letter

**To the Editor:** Schistosomiasis, known since ancient times, is caused by blood flukes (Trematoda: Schistosomidae). It is a major communicable disease with public health and socioeconomic effects in the developing world (*1*). Among parasitic diseases, schistosomiasis ranks second only to malaria with regard to the number of persons infected and at risk. The life cycle of schistosomes is complex, requiring specific freshwater snails as intermediate hosts for larvae development and multiplication. Among *Schistosoma* species that affect humans, *Schistosoma mansoni* is the most likely to invade new areas mainly because of the adaptability and invasiveness of its intermediate host, *Biomphalaria* snails. Natural populations of these snails are usually found in tropical standing water or freshwater in South America and Africa, but they also reach 30° latitude in subtropical areas (*1*,*2*). Many species of these red-blooded planorbid snails (Gastropoda: Basommatophora) are able to survive a long time when removed from their freshwater habitat (*1*). Of the 34 *Biomphalaria* species, 4 (*B. glabrata, B. pfeifferi, B. straminea*, and *B. tenagophila*) have recently expanded their native ranges (*3*). They have been introduced to areas where other *Biomphalaria* species are endemic (e.g., Congo and Egypt) or to subtropical zones that have no frost period (Texas, Louisiana, Florida, Hong Kong) (*3*,*4*). None of the known invasions, whether peripheral range expansion or long distance dispersal, reached the temperate zone. Spreading of the blood-fluke snails to schistosome-free areas may enable the parasite to colonize new habitats concurrently, expanding the potential area of clinical schistosomiasis.

We collected these snails in spring 2005, autumn 2006, and autumn 2007, near Răbăgani, Romania, Eastern Europe (46°45′1.3′′N, 22°12′44.8′′E) in a hypothermal spring. Water temperature was 25°C in the spring and 16°C–25°C, gradually decreasing, along the brook course. In and beside an abandoned concrete pool next to the spring, we collected 100 shells and 34 living specimens that macroscopically resembled *Biomphalaria* spp. snails. All 16 dissected animals proved to be fully developed adults, according to the maturity of their genital organs (Figure). Using available identification keys (*5*), we tentatively identified these snails as *B. tenagophila.* Voucher specimens have been deposited in the Hungarian Natural History Museum (accession nos. HNHM96857 and HNHM95433).

**Figure:**
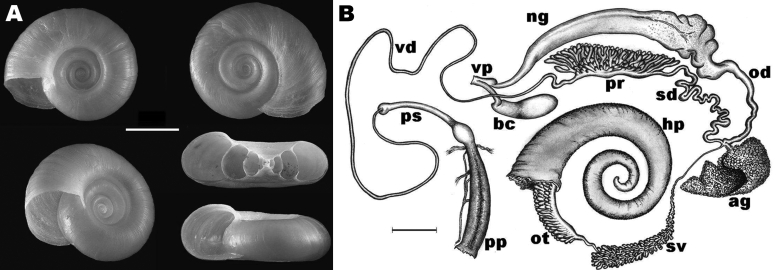
A) Shell morphology of *Biomphalaria tenagophila* snail from Romania. Diameter of the shell was 10–14 mm. The sinistrally coiled, flat shells are yellow-brown, discoidal, deeply and symmetrically biconcave, and consist of 5 or 6 slowly increasing whorls. The last whorl is rounded; the intermediate whorls are slightly angled on the left side. The aperture is circular or slightly ovate and angled toward the left side of the shell (i.e., toward the upper surface on the bottom right shell). Fine, parallel, rib-like transverse lines can be seen on the outer surface of the whorls. A series of photographs were prepared by focusing on different levels of the structure and these were combined by CombineZ5 (www.hadleyweb.pwp.blueyonder.co.uk), using “do combine” and “do average and filter” commands. Scale bar = 3 mm. B) Reproductive system of *B. tenagophila* snail from Romania; ag, albumin gland; bc, bursa copulatrix; hp, distal part of the hepatopancreas; ng, nidamental gland; od, oviduct; ot, ovotestis; pp, preputium; pr, prostate; ps, penis sheath; sd, spermiduct; sv, seminal vesicles; vd, vas deferens; vp, vaginal pouch. *B. tenagophila*–specific characteristics (*5*): >200 diverticulae of the ovotestis; 7–11 main lobes of the prostate; and presence of vaginal pouch. Scale bar = 1 mm.

DNA was extracted from the foot muscles of 3 specimens by using QIAamp DNA Mini Kit (QIAGEN, Hilden, Germany). For amplification of the partial mitochondrial 16S ribosomal RNA gene, we used a PCR with primers 16Sar and 16Sbr (*6*). Nucleotide sequences were determined in both directions. PCR products of ≈430 bp were detected from all 3 samples. Automatic cycle sequencing of the randomly selected amplicon (GenBank accession no. EU069412) showed 99.74% similarity to *B. tenagophila* (AF449615, Brazil).

Our morphologic, anatomic, and molecular data unambiguously prove the occurrence of *B. tenagophila* snails in Romania. *B. tenagophila* snails had been found earlier (in 2004) at this location but had presumably been misidentified as dwarf specimens of a common European species, *Planorbarius corneus* (*7*). Consequently, *B. tenagophila* snails have been not only introduced, but also established in Răbăgani, representing the furthest self-sustaining population of this species from the equator.

*B. tenagophila* is a new species for the European fauna. It could represent a founder population of unknown origin for further spread into Europe, which might easily be accomplished by migrating birds or more likely by plants used in aquariums (*3*). Although no trematode larvae were detected in the observed specimens, clinical schistosomiasis can be imported by immigrants or tourists into Europe, as has been reported in Romania and neighboring Hungary (*8*,*9*). If eggs were released in feces of humans infected with the blood flukes, they could hatch in the environment and the larvae could develop to an infective stage in these snails. The observed local cultural and social factors involving natural water (washing clothes, bathing) in Răbăgani where *B. tenagophila* have been found may also increase the chance of human infection.

We believe that *B. tenagophila* in Europe, together with the global climate change and a possible encounter of these snails with schistosomes, could pose a public health risk. Measures must be taken to prevent the spread of this species into European freshwater. Chemical control is not possible in Răbăgani because it is an area where other rare and endangered snail species are protected (*7*). Therefore, the manual collection and removal of all the *B. tenagophila* specimens in the area seems to be the only possibility for eradication, which might remain in effect for years. To avoid similar establishments, we suggest regular malacologic and parasitologic surveillance of at least the thermal and hypothermal water bodies for these tropical invaders around European settlements.
